# Identifying Thoracic Malignancies Through Pleural Fluid Biomarkers: A Predictive Multivariate Model

**DOI:** 10.1097/MD.0000000000003044

**Published:** 2016-03-11

**Authors:** José M. Porcel, Aureli Esquerda, Montserrat Martínez-Alonso, Silvia Bielsa, Antonieta Salud

**Affiliations:** From the Pleural Medicine Unit (JMP, SB); Departments of Internal Medicine, Laboratory Medicine (AE); Biostatistics (MMA); and Oncology-Hematology (AS), Arnau de Vilanova University Hospital, Biomedical Research Institute of Lleida, Lleida, Spain.

## Abstract

The diagnosis of malignant pleural effusions may be challenging when cytological examination of aspirated pleural fluid is equivocal or noncontributory. The purpose of this study was to identify protein candidate biomarkers differentially expressed in the pleural fluid of patients with mesothelioma, lung adenocarcinoma, lymphoma, and tuberculosis (TB).

A multiplex protein biochip comprising 120 biomarkers was used to determine the pleural fluid protein profile of 29 mesotheliomas, 29 lung adenocarcinomas, 12 lymphomas, and 35 tuberculosis. The relative abundance of these predetermined biomarkers among groups served to establish the differential diagnosis of: malignant versus benign (TB) effusions, lung adenocarcinoma versus mesothelioma, and lymphoma versus TB. The selected putative markers were validated using widely available commercial techniques in an independent sample of 102 patients.

Significant differences were found in the protein expressions of metalloproteinase-9 (MMP-9), cathepsin-B, C-reactive protein, and chondroitin sulfate between malignant and TB effusions. When integrated into a scoring model, these proteins yielded 85% sensitivity, 100% specificity, and an area under the curve (AUC) of 0.98 for labeling malignancy in the verification sample. For lung adenocarcinoma–mesothelioma discrimination, combining CA19-9, CA15-3, and kallikrein-12 had maximal discriminatory capacity (65% sensitivity, 100% specificity, AUC 0.94); figures which also refer to the validation set. Last, cathepsin-B in isolation was only moderately useful (sensitivity 89%, specificity 62%, AUC 0.75) in separating lymphomatous and TB effusions. However, this last differentiation improved significantly when cathepsin-B was used with respect to the patient's age (sensitivity 72%, specificity 100%, AUC 0.94).

In conclusion, panels of 4 (i.e., MMP-9, cathepsin-B, C-reactive protein, chondroitin sulfate), or 3 (i.e., CA19-9, CA15-3, kallikrein-12) different protein biomarkers on pleural fluid samples are highly discriminative for signaling a malignant versus tuberculous effusion, or lung adenocarcinoma versus mesothelioma, respectively. Cathepsin-B could also be helpful in establishing the presence of a lymphomatous effusion versus that of TB, if the patient's age is simultaneously taken into consideration.

## Abstract 2

Supplemental Digital Content is available in the text

## INTRODUCTION

Malignant pleural effusions are most quickly and easily diagnosed through a pleural fluid cytological examination. However, the cytological yield depends on many factors and is reported to be a maximum of 60%, although it is much lower in mesothelioma and squamous cell lung cancer.^1^ Therefore, the search for pleural fluid biomarkers of malignancy with the aim of avoiding further unnecessary invasive procedures or selecting those patients who may benefit the most from them is an ongoing quest.

To discriminate between malignant and tuberculous (TB) effusions may be difficult. First, because the biochemical characteristics of both malignant and TB pleural fluids are similar in that they are usually lymphocytic exudates. And second, pleural fluid adenosine deaminase (ADA), which is >35 to 40 U/L in 92% of TB effusions, has been reported to be falsely elevated in 10% of malignancies in general, and nearly 60% of lymphomas in particular.^2^ Thus, separating TB and lymphomatous effusions may pose a particular challenge and often requires immunophenotyping of lymphocytes into B- and T-cell subgroups (a predominance of small T-cells usually represents a benign process such as TB). Also demanding attention is the fact that when malignant cells are observed in pleural fluid, immunocytochemical studies of varying complexity are often necessary to distinguish between adenocarcinoma and mesothelioma.^3^ The underlying reason is that overlapping cytomorphological features of adenocarcinoma, reactive mesothelial cells, and malignant mesothelioma exist.

According to the preceding text, 3 potentially challenging scenarios could be envisioned in clinical practice, namely the differentiation of malignant and TB effusions, lung adenocarcinomas from mesotheliomas, and lymphomas with falsely elevated ADA from TB. In this study, we took advantage of protein microarray technologies applied to pleural fluid specimens for accomplishing these tasks. Initially, we searched for the most accurate protein combinations from a commercially available microarray biochip comprising 120 different proteins. Then, the results were validated in an independent population by using more clinically accepted protein methodologies.

## SUBJECTS AND METHODS

### Study Population

We have been maintaining a pleural fluid database and biobank of fluid specimens from every patient subjected to a diagnostic thoracentesis at the Arnau de Vilanova University Hospital (Lleida, Spain) for the last 18 years. All tapped pleural fluids are collected into EDTA tubes, centrifuged at 4°C, and the supernatant is frozen and stored at −80°C until assayed. For the current investigation, we used a computer-generated stratified randomization of effusions to initially select 78 samples, collected from 2001 to 2011 in our hospital, with the following etiological distribution: 30 lung adenocarcinomas, 12 lymphomas with high pleural fluid ADA levels, and 36 TB. Due to the low prevalence of mesotheliomas in the immediate local area, 30 such neoplasms originating from a different Spanish center (Hospital Universitario Virgen del Rocío, Sevilla, Spain) were incorporated into this derivation set. Each pair of independent samples of mesothelioma, lung adenocarcinoma, lymphoma, and TB was age- and sex-matched for comparisons. An independent randomly selected population of 30 lung adenocarcinomas, 20 mesotheliomas, 34 TB, and 18 lymphomas from our biobank served to validate the results of the protein microarrays applied to the derivation set. All pleural fluids which were analyzed had been collected before the institution of any specific treatment, including either antituberculous drugs or chemotherapy. The study was performed following the TRIPOD (transparent reporting of a multivariate prediction model for individual prognosis or diagnosis) statement.^4^ Approval for the study was obtained from the local ethics committee (CEIC, Arnau de Vilanova University Hospital, ID 764).

### Diagnostic Criteria for Pleural Effusions

The diagnosis of malignant effusions, whether mesotheliomas, adenocarcinomas, or lymphomas, was based on the demonstration of malignant cells in pleural fluid or biopsy specimens by an experienced pathologist. Specifically, an adequate tissue biopsy sample with the use of appropriate immunohistochemistry panels (i.e., at least 2 positive mesothelial markers and 2 negative adenocarcinoma markers) was required for confirming the diagnosis of mesothelioma.^3^ Commonly used immunohistochemical stains included mesothelin, cytokeratin 5/6, Wilms tumor-1 (mesothelioma markers); calretinin (mesothelial cell marker); epithelial membrane antigen (marker of malignancy, either mesothelioma, or adenocarcinoma); desmin (marker of reactive mesothelium); and thyroid transcription factor-1, napsin A and carcinoembryonic antigen (markers of adenocarcinoma). Additional stainings were performed in equivocal cases. TB pleuritis was diagnosed if Lowenstein–Jensen cultures of pleural fluid, sputum or pleural biopsy tissue samples were positive, or parietal pleural biopsies showed granulomas (i.e., confirmed TB cases), or an exudative lymphocytic effusion with high ADA levels (>35 U/L) cleared in response to anti-TB therapy in the absence of any other obvious explanation for the pleural fluid accumulation (i.e., probable TB cases).

### Microarray Protein Analyses

Pleural fluid samples from the derivation set were tested with the Whatman Schleicher & Schuell serum biomarker chip (Whatman GmbH, Dassel, Germany), according to the manufacturer's instructions. In brief, the chip consists of a single antibody capture array built on the FAST Slide dual pad platform. Each slide has 2 identical arrays of 120 antibodies printed in triplicate, which represent a myriad of biomarkers associated with human cancer. The original antibody chip was modified by replacing the free form of the prostate-specific antigen with mesothelin (Table 1). A pleural fluid volume of 1.5 to 6 μL was used to achieve a total protein amount of 100 to 120 μg in the slide chambers for incubation and labeling reaction. Two-color fluorescent detection (Biotin-ULS and Fluorescein-ULS) enabled spot intensity mapping of the relative abundance of the 120 proteins between 2-paired samples, one from the malignant disease (cases) and the other from TB (controls). Spot intensities were displayed using ArrayVision FAST software, and a bioinformatic analysis converted fluorescent data into numerical values which represented the abundance of antigen in cases relative to controls. The ratio of the average spot intensities for each paired samples was plotted on a Log 2 scale to reveal the over- and under-abundance pleural fluid biomarkers in cancer patients as compared to TB or, where appropriate, in lung adenocarcinoma versus mesothelioma. The first step of sample labeling with Biotin and Fluorescein was performed in our center. Labeled microarrays were then sent for both scanning (Axon GenePix 4200A, Molecular Devices Corporation, Sunnycale, CA) and data analysis in blinded fashion to the Maine Manufacturing LLC company (Sanford, ME).

**TABLE 1:**
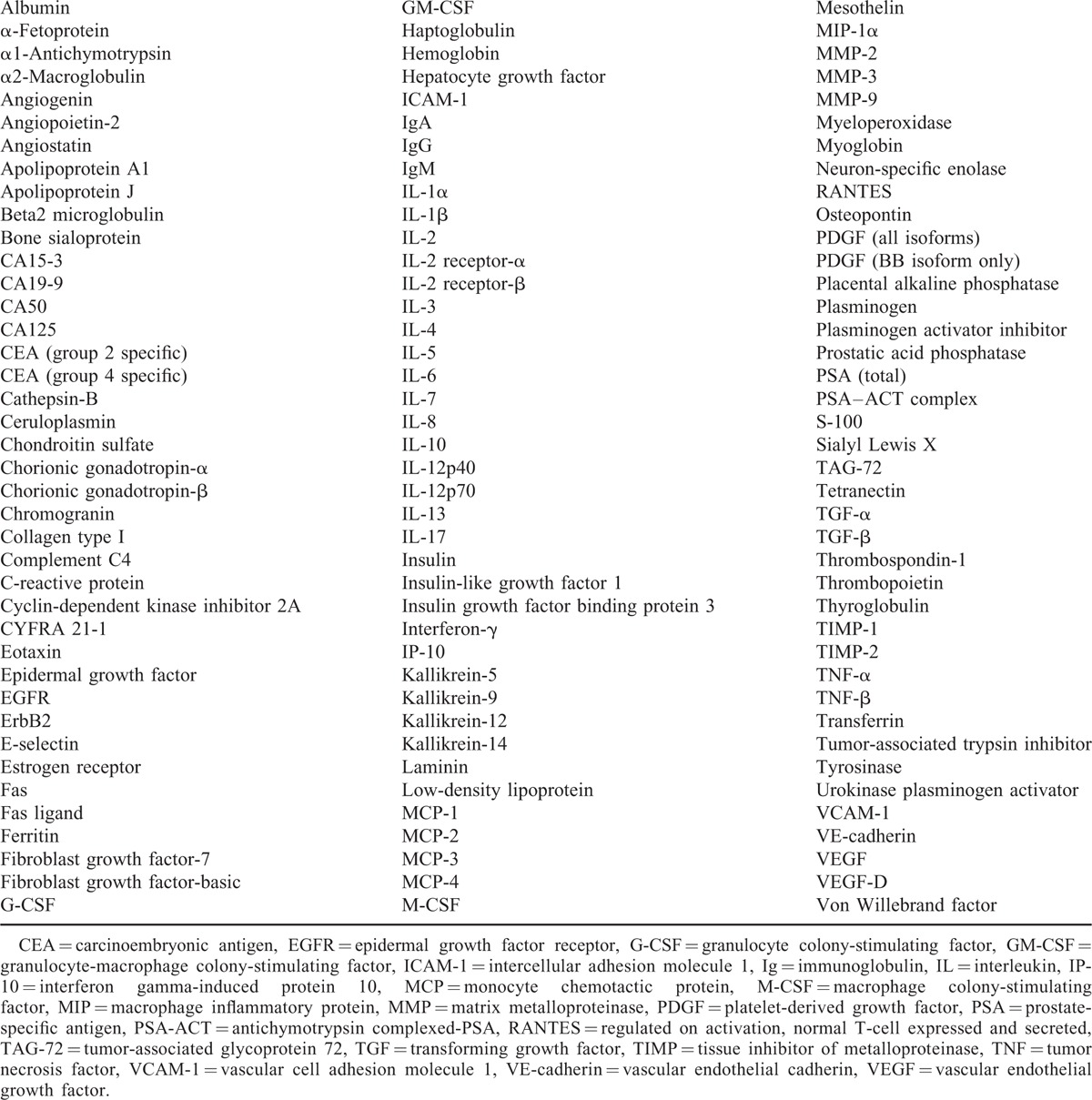
Biomarker-Specific Antibodies Evaluated in the Derivation Sample

### Other Pleural Fluid Measurements

Pleural fluid ADA levels were determined by using an automated ultraviolet kinetic assay (Roche Diagnostics, Barcelona, Spain). Nine biomarkers, which were selected as potentially discriminatory from the derivation sample, were measured either by enzyme-linked immunosorbert assays (human cathepsin-B, human kallikrein-12, human condroitin sulfate, human matrix metalloproteinase 3 [MMP-3], human matrix metalloproteinase 9 [MMP-9], and human angiostatin; Cusabio Biothech), a particle-enhanced immunoturbidimetric assay (C-reactive protein; Roche Diagnostics GmbH, Mannheim, Germany), or electro-chemiluminiscence immunoassays (CA15-3 and CA19-9; Roche Diagnostics GmbH, Mannheim, Germany) in the pleural fluid of an independent population.

### Statistical Analysis

Sample size calculation for the derivation set was based on a previously proposed method for bilateral contrasts, aiming for a statistical power of at least 80% and a false discovery rate of 5%.^5^

Comparative analyses of the pleural fluid protein profiles of malignant (i.e., adenocarcinomas plus mesotheliomas) versus TB effusions, lung adenocarcinomas versus mesotheliomas, and lymphomas versus TB (both exhibiting high fluid ADA levels) were carried out. The results of a Pearson correlation, which measured the linear relationship of the protein profiles among groups, were graphically displayed using a heatmap. Differences in protein expression between groups were analyzed by the Wilcoxon test, controlling the false discovery rate (FDR) at *P* < 0.05. Then, tree-based algorithms were generated using supervised Random forests (i.e., an ensemble classifier using many decision tree models) multivariate methodologies to lessen potential biomarker candidate proteins,^6^ and Gini index^7^ as an impurity-based criterion for calculating a node. These classification algorithms were built through recursive partitioning or conditional inference.

The chosen protein biomarkers from the training set were subsequently validated for the 3 comparisons, using the likelihood ratio (LR) test to compare the goodness of fit between 2 models. First, logistic regression models were fitted and ROC curves were used to calculate optimal cutoff values for individual markers while searching for, whenever possible, a 100% specificity. Then, a scoring system was devised in which weight values to each biomarker were assigned proportionally to the magnitude of the logistic equation's coefficients. Finally, the discriminative properties of the scoring model (i.e., sensitivity, specificity, accuracy, LR positive, and negative) were estimated. Whenever specificity was 100%, it was statistically necessary to artificially add 1 case to the false positive cell of a 2 × 2 table in order to enable the calculation of the LR positive. The addition of sex and/or age to improve the discrimination capability of protein-based scores was also assessed. All analyses were performed with the R software for statistical computing (http://www.r-project.org/).

## RESULTS

### Characteristics of the Study Population

Three cases (1 each mesothelioma, lung adenocarcinoma, and tuberculosis) were excluded from the derivation analysis because the biochip slides were accidentally broken during the labeling procedure. Demographic and pleural fluid data of the 105 patients from the derivation sample and 102 patients from the validation sample are presented in Table 2. Both groups were homogeneous, though there were some minor statistical differences in the baseline characteristics between them. Specifically, the median age of the TB derivation sample surpassed that of the validation sample (44 vs 31 years, *P* < 0.001), whereas less than one-third of lymphomatous effusions from the validation population had high pleural fluid ADA levels as compared to 100% of the derivation population. The cause for this latter discrepancy was the difficulty in recruiting new patients with such a relatively infrequent cause of pleural effusion which, in addition, had to exhibit elevated ADA concentrations.

**TABLE 2:**
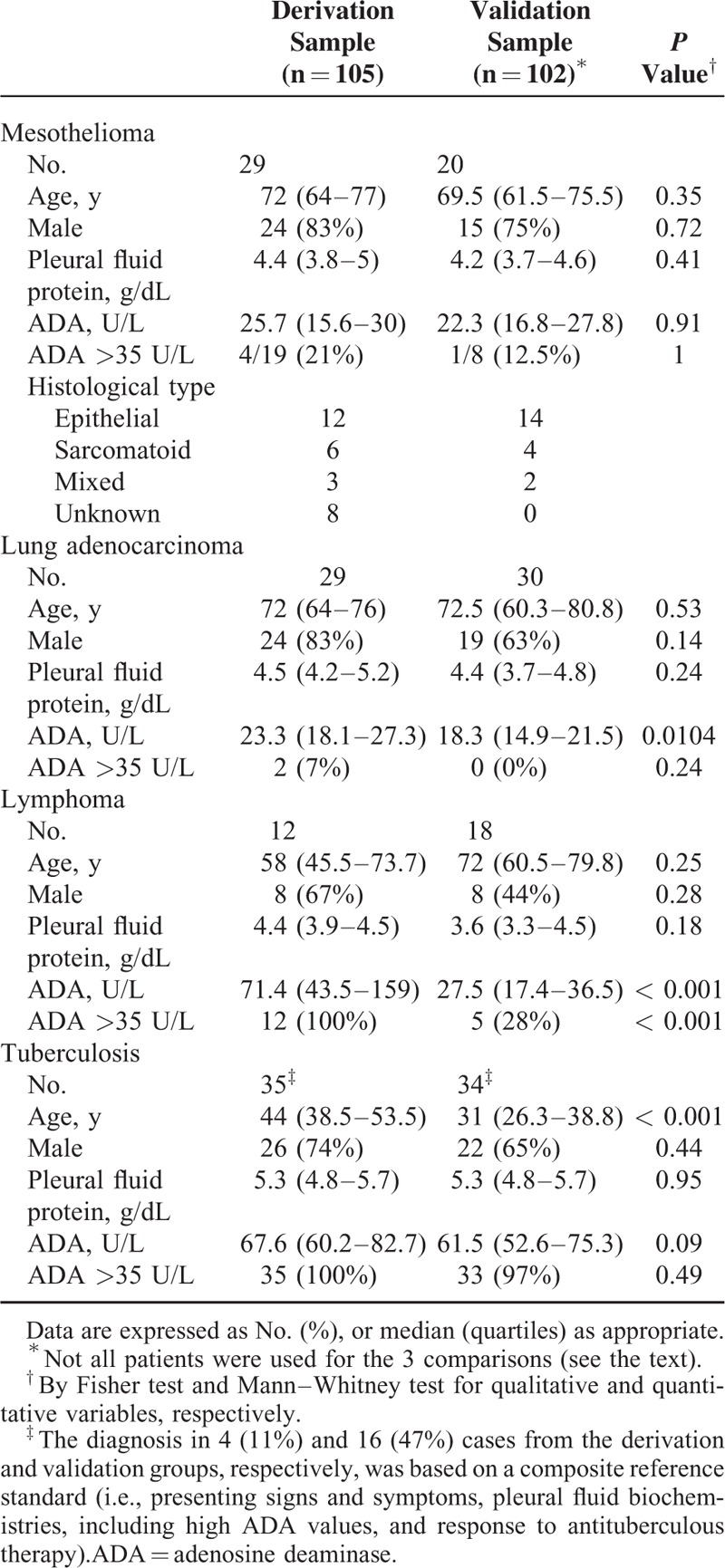
Baseline Characteristics of the Study Population

### Differential Protein Expression Between Groups in the Derivation Sample

**FIGURE 1:**
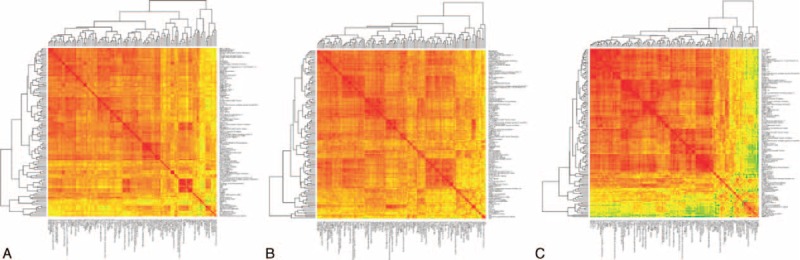
Heatmaps with linear correlations of proteins for the comparisons between malignant and TB effusions (A), lung adenocarcinoma versus mesothelioma (B), and lymphoma versus TB (C). Red corresponds to positive correlations (*r* = 1), green to negative correlations (*r* = −1), and yellow to lack of correlations (*r* = 0).TB = tuberculosis.

**FIGURE 2:**
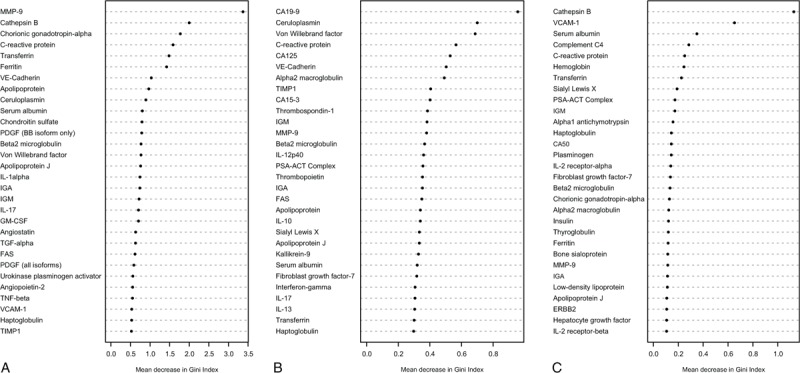
Supervised random forest for the differentially expressed proteins among the 3 comparisons (A, malignant vs TB; B, lung adenocarcinoma vs mesothelioma; C, lymphoma vs TB).TB = tuberculosis.

After graphically expressing protein correlations for the 3 groups of paired comparisons in a heat map matrix (Figure 1), differentially expressed proteins were determined using the FDR approach and random forest methodologies as a tree-based method (Figure 2).

In the first comparison, proteins which most contributed to discriminate between 58 malignant (of which half were lung adenocarcinomas and half mesotheliomas) and their corresponding paired TB effusions were MMP-9, cathepsin-B, C-reactive protein, angiostatin, and chondroitin sulfate (Figure 2A). A constructed tree with these respective proteins running down the tree root, from the base to terminal nodes, had a sensitivity of 95% (95% CI 86–99%), and a specificity of 84.7% (95% CI 73–92.8%) for labeling malignancy.

In the second comparison, a classification tree which sequentially included the proteins CA19-9, CA15-3, MMP-3, and kallikrein-12 (Figure 2B) yielded a sensitivity of 83% (95% CI 64.2–94.2%) and a specificity of 89.7% (95% CI 73–98%) for separating 29 lung adenocarcinomas from 29 mesotheliomas.

Finally, in the third, cathepsin-B (Figure 2C) was able to discriminate 12 lymphomas with high ADA levels from 12 TB with 100% sensitivity (95% CI 73.5–100%) and 83.3% specificity (95% CI 51.6–98%). This paper does not include a visual depiction for any of these algorithms from the training set because optimal cutoff points for each participating protein were expressed as signal intensity units, which are of cumbersome interpretation for the practicing physician.

### Accuracy of Different Protein Profiles in the Validation Sample

**FIGURE 3:**
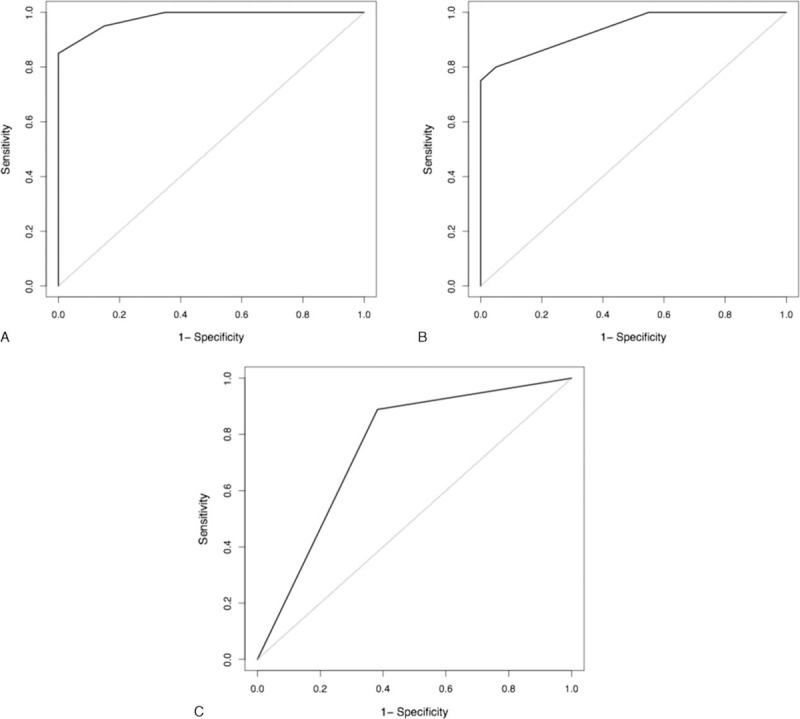
Receiver operating characteristic curves for the differentially expressed proteins among the 3 comparisons (A, malignant vs TB; B, lung adenocarcinoma vs mesothelioma; C, lymphoma vs TB). TB = tuberculosis.

A different set of 20 malignant (10 lung adenocarcinomas, 5 mesotheliomas, and 5 lymphomas) and 20 TB effusions was used to validate the usefulness of the previously selected proteins in the microarray assays, but this time using commercially available methodologies. We established a score system as follows: MMP-9 < 15.5 ng/mL (3 points), chondroitin sulfate >1.25 ng/mL (3 points), cathepsin-B <0.42 ng/mL (2 points), and C-reactive protein <12.5 mg/L (1 point). The model, which excluded angiostatin as a significant contributor, had an area under the curve (AUC) of 0.98 (95% CI 0.95–1) (Figure 3A). A score ≥6 indicated malignancy with 85% sensitivity (95% CI 62.1–96.8%), 100% specificity (95% CI 83.2–100%), LR positive of 17.9 (95% CI 2.6–121.9), and LR negative of 0.15 (95% CI 0.05–0.43) (Table 3).

**TABLE 3:**
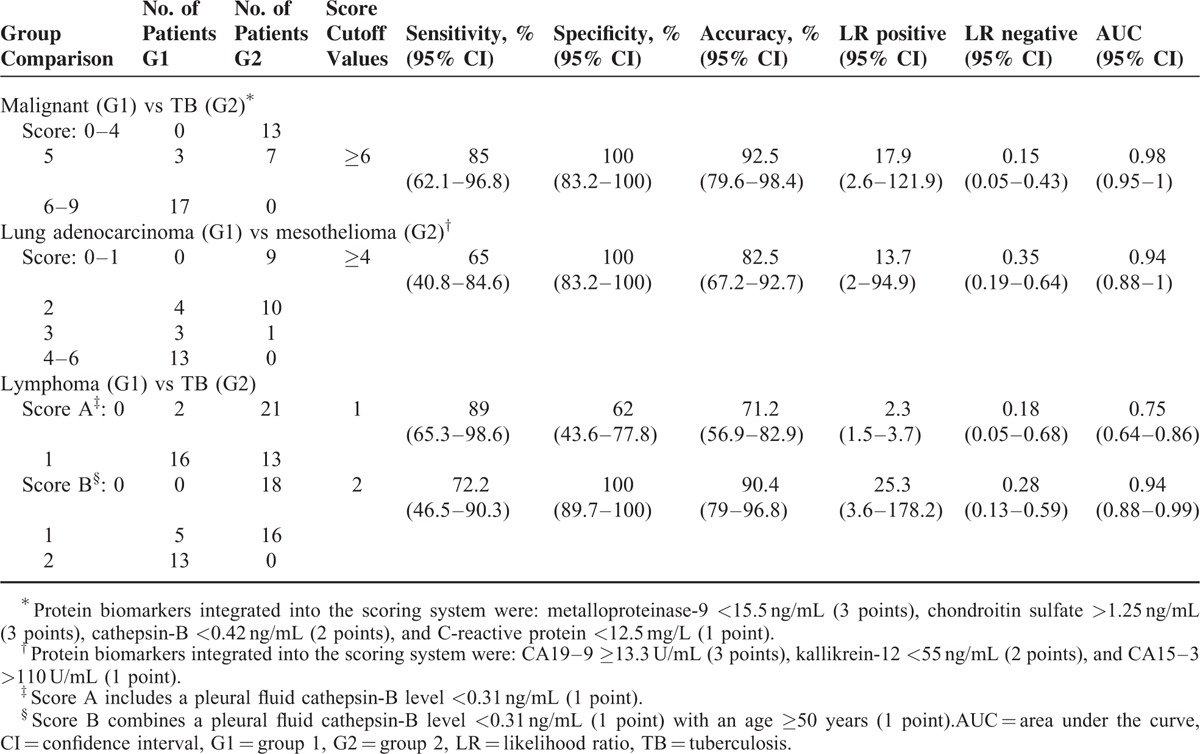
Discriminative Properties of the Scoring Systems in the Validation Set

Similarly, 20 lung adenocarcinomas and 20 mesotheliomas were tested for the expression of the pleural fluid proteins CA19-9, CA15-3, kallikrein-12, and MMP-3. The last was dismissed by the following best scoring model, which had an AUC of 0.94 (95% CI 0.88–1) (Figure 3B): CA19–9 ≥13.3 U/mL (3 points), kallikrein-12 <55 ng/mL (2 points), and CA15-3 >110 U/mL (1 point). A score ≥4 separated lung adenocarcinomas from mesotheliomas with 65% sensitivity (95% CI 40.8–84.6%), 100% specificity (95% CI 83.2–100%), LR positive of 13.7 (95% CI 2–94.9), and LR negative of 0.35 (95% CI 0.19–0.64) (Table 3).

Finally, a pleural fluid cathepsin-B level <0.31 ng/mL yielded 89% sensitivity (95% CI 65.3–98.6%), 62% specificity (95% CI 43.6–77.8%), LR positive of 2.3 (95% CI 1.5–3.7), and LR negative of 0.18 (95% CI 0.05–0.68) for separating 18 lymphomas from 34 TB (Table 3). The AUC was 0.75 (95% CI 0.64–0.86) (Figure 3C). In the first 2 comparisons (malignant vs TB, lung adenocarcinoma vs mesothelioma), the incorporation of demographic data (age, sex) into the fluid biomarker panel did not improve the operating characteristics of the scoring models. However, this was not true for the discrimination of lymphoma versus TB (see Table, supplemental content which illustrates the corresponding LR tests). In particular, patients who scored 2 points, in which 1 point each was assigned to both pleural fluid cathepsin-B concentrations <0.31 ng/mL and age ≥50 years, were most likely to have lymphomas rather than TB (sensitivity 72.2%, 95% CI 46.5–90.3%; specificity 100%, 95% CI 89.7–100%; LR positive 25.3, 95% CI 3.6–178.2; LR negative 0.28, 95% CI 0.13–0.59; and AUC 0.94, 95% CI 0.88–0.99).

The previous 3 scoring systems were obtained after dichotomizing the protein biomarker expressions, which showed better discriminating properties than the use of continuous protein expressions (see Figures 1–3, supplemental content which illustrates calibration and ROC plots for both binary and continuous approaches).

## DISCUSSION

In the current investigation, we used a protein chip array technology to discover candidate pleural fluid biomarkers for malignancy. As the biomarker chip was simply a method to ascertain protein abundance changes between biological samples of different etiologies, we subsequently intended to provide protein quantification by using commercially available methodologies in an independent population.

First, it was found that the combined measurement in the pleural fluid of MMP-9, chondroitin sulfate, cathepsin-B, and C-reactive protein was highly accurate for discriminating between malignant and benign (TB) effusions. When integrated into a simple scoring system, these proteins yielded 85% sensitivity and 100% specificity for labeling malignancy. Such impressive figures contrast with the reported 60% sensitivity of the cytological examination (the diagnostic gold standard) while greatly surpassing that of classical tumor marker panels (54% in a large single study in which maximal specificity was attained).^8^ Except for chondroitin sulfate, the remaining 3 biomarkers were underexpressed in malignant fluids as compared with TB. MMP-9 is involved in the development of TB granulomas and, therefore, TB effusions contain higher concentrations when compared with malignant effusions and transudates.^9–11^ Cathepsin-B belongs to a family of lysosomal cysteine proteases and plays a role in intracellular proteolysis. It is difficult to find a satisfactory explanation for why its expression was downregulated in malignant effusions when compared with TB. However, in some experimental models, significant amounts of cathepsin-B were observed in washing fluids of subcutaneous inflammatory foci of *Mycobacterium tuberculosis*-induced hypersensitivity reactions.^12^ C-reactive protein, an acute-phase protein widely used as a marker of inflammation, may be a diagnostic aid in the differential diagnosis of pleural effusions. Previous studies have consistently reported that levels of C-reactive protein in pleural fluids are 2 to 4 times higher in TB pleuritis than malignant effusions;^13–15^ the more intense inflammation in the former as compared with the latter probably accounts for this difference. For example, in the largest study which comprised of 55 TB and 60 malignant effusions, the mean pleural fluid C-reactive protein levels were 54.6 mg/L and 12.6 mg/L respectively,^13^ which is consistent with our results. Finally, although chondroitin sulfate on the tumor cell surface and in the extracellular matrixes is related to metastatic potential and facilitates tumor invasion, it has never been tested before in pleural fluid samples.

A second clinical challenge is the cytomorphological differentiation between adenocarcinoma and mesothelioma. In clinical practice, this discrimination requires the application of a number of immunocytochemical panels on pleural fluid cell blocks or, even better, on pleural biopsy specimens. We found that high pleural fluid concentrations of 2 classical tumor markers (i.e., CA15-3 and CA19-9) simultaneously, or alternatively an elevated CA19-9 along with low kallikrein-12 concentrations, pointed to a metastatic lung adenocarcinoma rather than mesothelioma, with 65% sensitivity and 100% specificity. In a recent meta-analysis of 49 studies, pleural fluid CA15-3 and CA19-9 exhibited 50.7% and 37.6% pooled sensitivities, respectively, and 98% specificities for diagnosing malignant effusions.^16^ Moreover, fluid concentrations may be significantly different among various tumor types. In this sense, Wang et al measured the pleural fluid levels of 5 tumor markers, including CA15-3 and CA19-9, in 251 patients with different tumors. Significantly higher levels of CA15-3 (138.6 U/mL vs 21.3 U/mL) and CA19-9 (516.6 U/mL vs 181.5 U/mL) were found in 128 lung adenocarcinoma cases than in 18 mesotheliomas.^17^ Kallikreins are a subgroup of serine proteases which have been implicated in carcinogenesis. Tissue expression of kallikreins has been demonstrated in pleural mesothelioma,^18^ yet explanations for their levels and significance in malignant pleural fluids is lacking. The observation that soluble mesothelin, the reference biomarker of mesothelioma,^19^ was not found to be differentially expressed in mesotheliomas when compared to lung adenocarcinomas may be explained by the use of a different platform assay and the fact that a quarter of advanced lung adenocarcinomas express high levels of mesothelin.^20^

Regarding the last issue, namely the differentiation between lymphomatous and tuberculous effusions, cathepsin-B alone, which promised to be a useful marker in the derivation sample, failed to confirm these expectations in the validation sample. It is probable that its combination with VACM-1, the second most powerful differentially expressed protein in the random forest analysis, would have increased discriminating capacity. It is certain that combining cathepsin-B with a simple clinical variable such as age did greatly enhance lymphoma-TB separation. This may have been because pleural TB typically affects younger patients as compared to lymphoma-associated effusions.^1,2,21^

During the last decade, the identification of novel biomarker candidate proteins of malignant effusions has been attempted using proteomic analysis. However, most studies lack adequate sample sizes, are heterogeneous in the comparison groups and the proteomic technologies applied, and generally do not validate their findings.^22^ The choice of an appropriate proteomic methodology depends on many factors, including the availability of resources and the number of samples to be processed. Rather than exploring putative biomarkers through complex or research-limited proteomic methodologies in a blinded strategy (i.e., the proteome to be investigated is not known),^23,24^ we made use of a biochip array which allowed us to determine multiple prespecified protein biomarkers (guided strategy) from a single sample. We selected commercially available technologies for both the biomarker chip and protein validation with the aim of increasing the reproducibility and clinical applicability of our research.

Several limitations of the present study should be addressed. First, a relatively small number of pleural fluid samples were tested, particularly from the lymphomatous etiology. In fact, we could not firmly verify any set of potential biomarkers for lymphoma-TB separation, unless used in combination with patient's age. Second, causes of benign effusions other than TB were not explored. Therefore, the claimed biomarkers’ accuracy may not be valid for separating malignant and non-TB effusions, yet TB is generally the most challenging differential diagnosis. Lastly, the reported biomarkers need to be explored and validated in new large independent populations with the techniques used in the verification stage, and also in a subgroup of cancer patients with falsely negative or misleading pleural fluid cytology.

## CONCLUSIONS

Our study demonstrated the diagnostic power of a new panel of biomarkers for the workup of pleural effusions, specifically for malignant-TB and lung adenocarcinoma-mesothelioma differentiations. Moreover, single fluid proteins in combination with clinical parameters such as age may help to further differentiate lymphomatous and TB effusions. Even though these panels showed maximal specificity, they are not intended to replace standardized diagnostic procedures such as pleural fluid cytology or biopsy for labeling malignant effusions. Rather, they may provide additional information when deciding whether or not to perform invasive procedures.

## Supplementary Material

Supplemental Digital Content
