# Supplementary material for: Identifying Thoracic Malignancies Through Pleural Fluid Biomarkers: A Predictive Multivariate Model
Source: Medicine (Baltimore). 2016 Mar 11;95(10):e3044. doi: 10.1097/MD.0000000000003044 (PMC4998909; doi:10.1097/MD.0000000000003044)
Supplement: Supplemental Digital Content [file medi-95-e03044-s001.pdf]

Fig. 1. Calibration & ROC plots of the predictive model for malignant vs. TB effusions

A. Based on dichotomized protein expressions

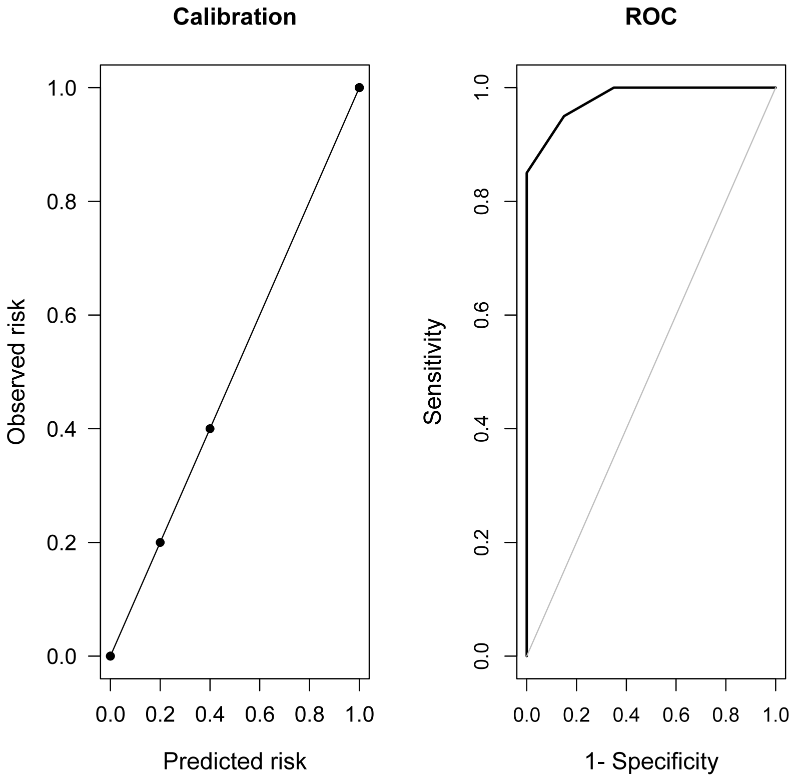

B. Based on continuous protein expressions

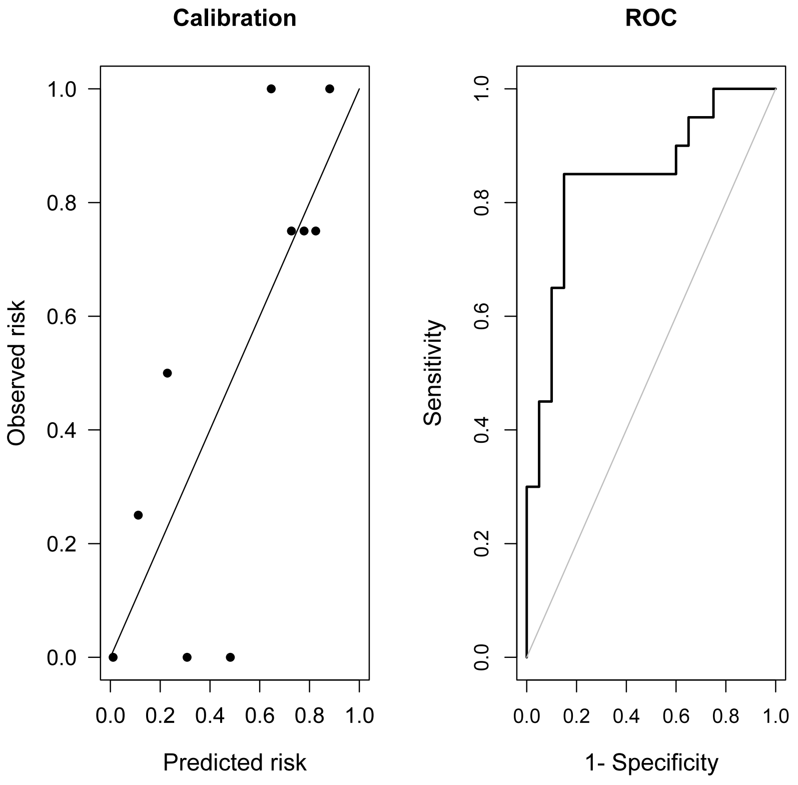

Fig. 2. Calibration & ROC plots of the predictive model for lung adenocarcinoma vs. mesothelioma

A. Based on dichotomized protein expressions

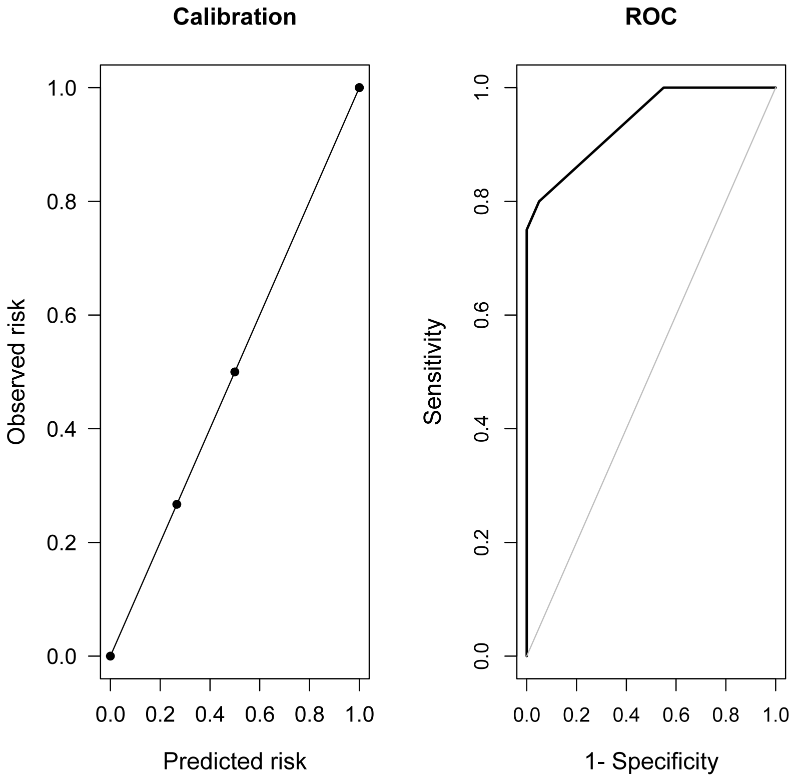

B. Based on continuous protein expressions

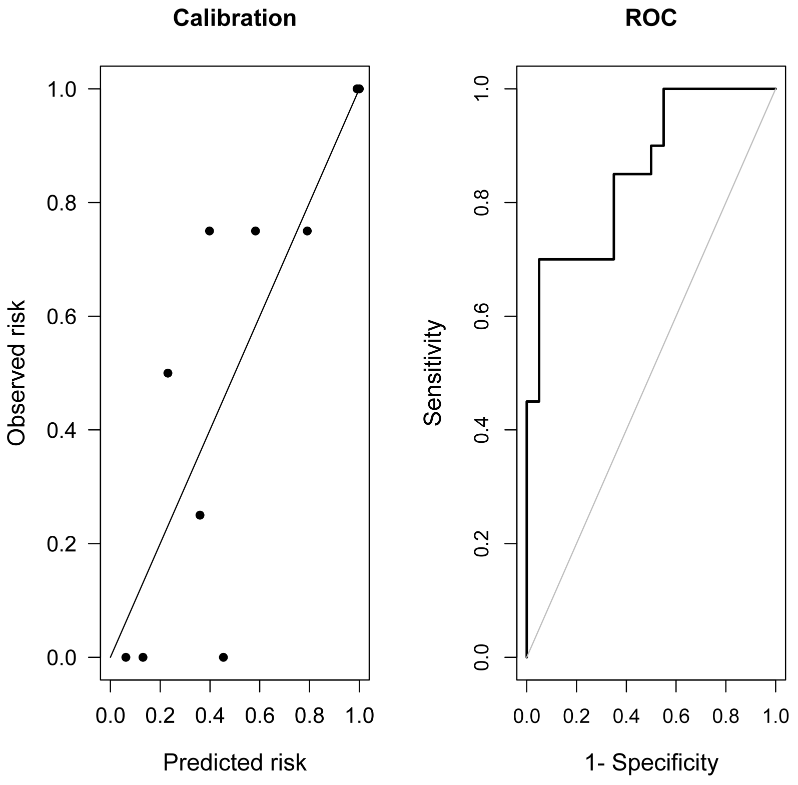

Fig. 3. Calibration & ROC plots of the predictive model for lymphoma vs. TB

A. Based on dichotomized protein expressions

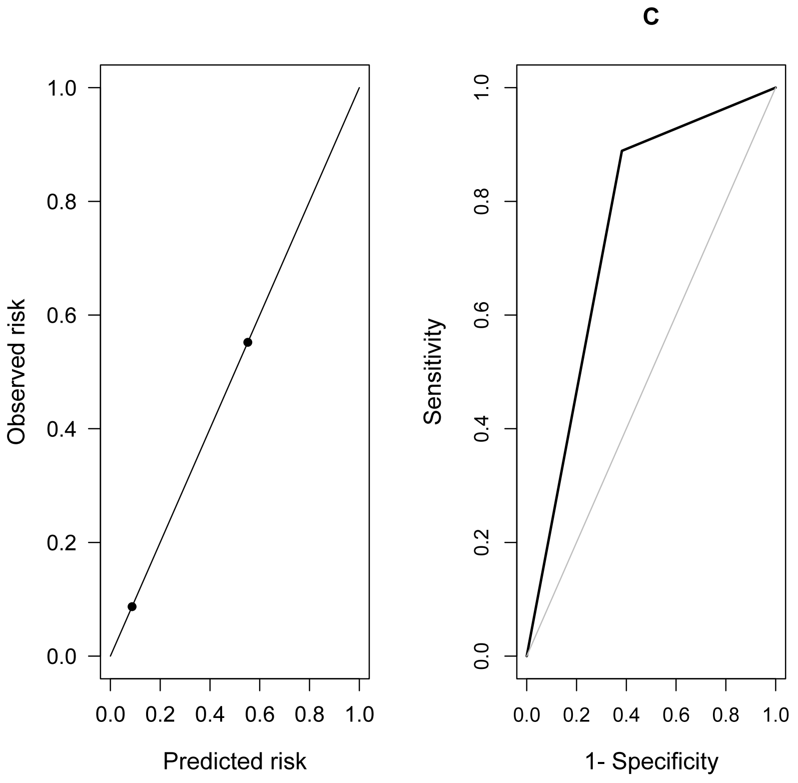

B. Based on continuous protein expressions

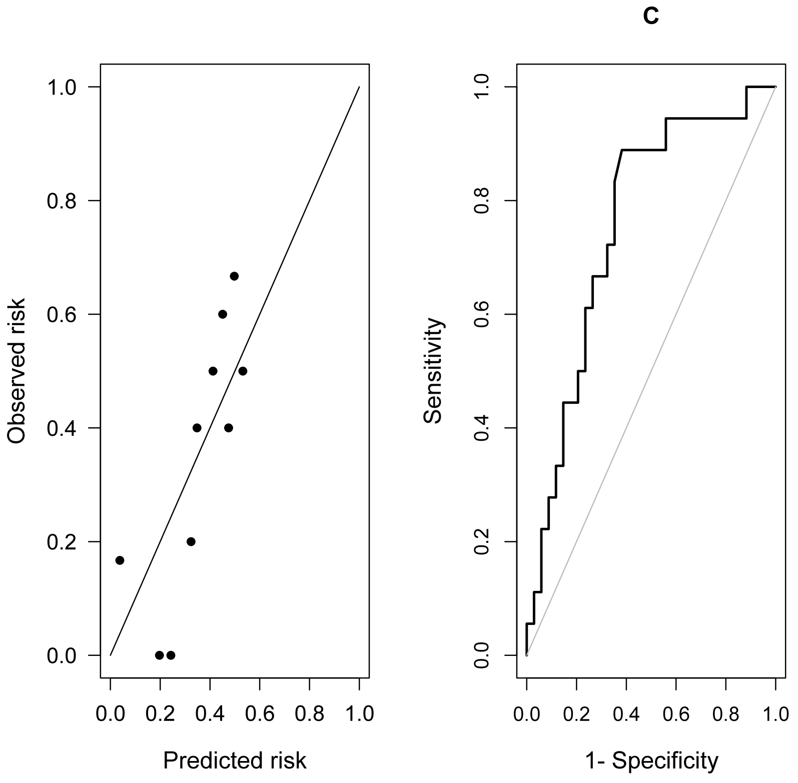

Table. Additional contribution of demographical variables (sex and age) to the proposed scoring systems

A. Malignant vs. TB effusions

Statistical significance of sex contribution

```
Analysis of Deviance (Likelihood ratio test)
                 Resid. Df Resid. Dev Df Deviance Pr(>Chi)
Score>=6                38 2.3206e-10
Score>=6 and Sex        37 2.3206e-10  1        0        1
```

Statistical significance of age contribution

```
Analysis of Deviance (Likelihood ratio test)
                 Resid. Df Resid. Dev Df Deviance Pr(>Chi)
Score>=6                38 2.3206e-10
Score>=6 and Age        37 2.3206e-10  1        0        1
```

B. Lung adenocarcinoma vs. mesothelioma

Statistical significance of sex contribution

```
Analysis of Deviance (Likelihood ratio test)
                 Resid. Df Resid. Dev Df Deviance Pr(>Chi)
Score≥4                 38     30.903
Score≥4 and Sex         37     30.527  1  0.37648   0.5395
```

Statistical significance of age contribution

```
Analysis of Deviance (Likelihood ratio test)
                 Resid. Df Resid. Dev Df Deviance Pr(>Chi)
Score≥4                 38     30.903
Score≥4 and Age         37     29.683  1   1.2204   0.2693
```

C. Lymphoma vs. TB

Statistical significance of sex contribution

```
Analysis of Deviance (Likelihood ratio test)
                 Resid. Df Resid. Dev Df Deviance Pr(>Chi)
Score=1                 50     53.482
Score=1 and Sex         49     51.580  1    1.902   0.1679
```

Statistical significance of age contribution

```
Analysis of Deviance (Likelihood ratio test)
                Resid. Df Resid. Dev Df Deviance  Pr(>Chi)
Score=1                50     53.482
Score=1 and Age        49     26.737  1   26.745 0.00000023
Score=1 and Age≥50     49     22.173  1   31.309 0.00000002
```
